# Establishment of hairy root lines and analysis of iridoids and secoiridoids in the medicinal plant *Gentiana scabra*

**DOI:** 10.1186/1999-3110-55-17

**Published:** 2014-02-02

**Authors:** Shih-Hung Huang, Rishi Kishore Vishwakarma, Tzu-Tai Lee, Hsiao-Sung Chan, Hsin-Sheng Tsay

**Affiliations:** 1 Department of Applied Chemistry, Chaoyang University of Technology, Taichung, Taiwan; 2 Department of Animal Science, National Chung Hsing University, Taichung, Taiwan; 3 Department of Agronomy, National Chung Hsing University, Taichung, Taiwan

**Keywords:** *Gentiana scabra*, Hairy root culture, Iridoids and secoiridoids, Plant growth regulators

## Abstract

**Background:**

*Gentiana scabra* is commonly known as ‘Longdan’ is an important herb in traditional Chinese medicines, commonly used for the treatment of inflammation, anorexia, indigestion and gastric infections. Iridoids and secoiridoids are main bioactive compounds which attributed to the pharmacological properties of this plant. The use of hairy root cultures as an excellent alternative for the production of pharmaceutically important metabolites in less time period with ensured quality of raw materials.

**Results:**

An efficient hairy root culture system of *Gentiana scabra* and influence of different plant growth regulators (PGRs) on the production of gentiopicroside, swertiamarin and loganic acid constituents were described. Leaf explants were infected with *Agrobacterium rhizogenes*, which induced hairy roots up to 21%. The transformed hairy root lines were confirmed by PCR using *rolB* and *rolC* gene-specific primers. Among various solid and liquid media, B5 liquid medium resulted maximum root biomass (36- fold higher) in 4-weeks. Quantitative analysis showed loganic acid was 6.6- fold higher in the presence of zeatin (1 mg/l) and gentiopicroside accumulation was 1.8- fold higher in the presence of naphthaleneacetic acid (NAA, 1 mg/l), as compared to the roots of plants grown in greenhouse. On the other hand, 1.4- and 2.5- fold higher gentiopicroside and swertiamarin were observed in the presence of 1.0 mg/l NAA as compared to commercial *Gentiana* herb No. 2. The result also showed iridoid and secoiridoid contents affected greatly by age, physiology and growing environment of the plant.

**Conclusions:**

The use of hairy root cultures is an excellent alternative to harvesting natural or in vitro grown plants to produce pharmaceutically important metabolites in less time with ensured quality.

**Electronic supplementary material:**

The online version of this article (doi:10.1186/1999-3110-55-17) contains supplementary material, which is available to authorized users.

## Background

Plant metabolites are affected by soil and climatic variations, thus their growth in controlled environment overcomes several of their production limitations. Tissue culture has also become an alternative way to obtain products when important methods or economic viability are challenged. Organized cultures, especially root cultures, can make a significant contribution to phytochemicals production. The neoplastic (cancerous) roots produced by *A. rhizogenes* infection are characterized by easy maintained, genetic stability, fast growth and growth in hormone-free media (Chandra and Chandra 2011). The greatest advantage of hairy roots is that they often exhibit similar or synthesized at levels higher than in untransformed tissue (Mannan et al. 2008). Hairy roots of goldenrod were induced infecting axgenic plants by *A. rhizogenes* A4 strain to produce allelopathic polyacetylene (Inoguchi et al. 2003). Hairy root cultures are also known to produce a spectrum of secondary metabolites that are not present in the parent plant (Aberham et al. 2011). Medicinal plants have been widely explored for hairy root culture and their secondary metabolites (Gupta et al. 2011; Wilczanska-Barska et al. 2012). Recently, biotransformation of coumarin glycosides by transgenic hairy roots of *Polygonum multiflorum* was reported using different substrates (Zhou et al. 2012).

The genus *Gentiana* comprised about 400 species which are widely distributed in temperate regions of Asia, Europe, the Americas, northwest Africa, eastern Australia and New Zealand (Georgieva et al. 2005; Zając and Pindel 2011). In Asia, the root of *Gentiana scabra* is commonly known as ‘Longdan’ in Chinese herbal medicines and has been used in the treatment of inflammation, anorexia, indigestion and gastric infections for over 2000 years (Tang and Eisenbrand 1992). The medicinal values of *Gentiana* spp. are extensive including anti-inflammatory, analgesic, antirheumatic, antipyretic, diuretic and hypoglycemic properties (Sezik et al. 2005; Chen et al. 2008; Wani et al. 2013). Chemical investigation of root extract of *Gentiana* spp. resulted in isolation of a series of iridoids, secoiridoids, xanthones and xanthone glycosides (Aberham et al. 2011). The gentiopicroside, swertiamarin and loganic acid are important active components used for gentian identification.

Several reports have documented successful inoculation of *Gentiana* species with *A. rhizogenes*, resulting in hairy root formation (Mugnier 1988; Tepfer 1990; Momčilović et al. 1997). However, most of them were mainly engrossed in hairy root development after *A. rhizogenes* transformation, and regeneration system (Hosokawa et al. 1997; Mishiba et al. 2006). Only a few studies focused on the secondary metabolite content analysis in hairy roots of *Gentiana* species, such as *G. macrophata* where richest gentiopicroside content (2.86%) was reported among the entire hairy root lines (Hayta et al. 2011).

In the present study induction process and characteristics of the hairy root lines from *G. scabra* has been described. The effects of different plant growth regulators (PGRs) on the hairy root growth and accumulation of loganic acid, gentiopicroside and swertiamarin in hairy roots were also investigated. Comparative analysis of iridoids and secoiridoids content was also performed with commercially available *G. scabra* herbs and uniformly grown greenhouse plants.

## Methods

### Plant materials and culture conditions

The plantlets of *G. scabra* were grown on half-strength Murashige-Skoog (MS) medium (Murashige and Skoog 1962) supplemented 0.1 mg/l indole-3-butyric acid (IBA), 3% sucrose and 0.3% gelrite. Uniform culture conditions were applied for all the experiments. The pH of the media was adjusted to 5.7 ± 0.1 before autoclaving. The media was autoclaved for 15 min at a pressure of 1.05 kg/cm^2^ at 121°C. Cultures were incubated at 25 ± 1°C under cool-white fluorescent light at 40 μmol/ m^2^s under 16-h day-periods for 6 weeks. The leaf explants were used for inoculation with *A. rhizogenes*.

### *Agrobacterium rhizogenes*-mediated hairy roots transformation and the time course of the study

The inoculation procedures were followed as described by Gupta et al. (2011) with slight modifications. The leaves of *in vitro* plantlets of *G. scabra* were cut into 0.25 cm^2^ pieces and used as an explant. The explants were pre-cultured on MS basal medium for 24 h prior to infection. *A. rhizogenes* strains ATCC15834 (Food Industry Development Institute, Taiwan) were grown overnight on BEP medium (beef extract and peptone) at 28°C and 180 rpm in the dark. *A. rhizogenes* were inoculated into fresh BEP media and grown for 48 h. Cells were harvested by centrifugation at 4000 rpm for 15 min and resuspended in liquid MS basal medium until OD_600_ reached 0.8-1.0. The pre-cultured explants were submerged into the bacterial suspension and acetosyringone was added to a final concentration of 100 μM, and incubated for 30 min in shaking condition. After blotting off the excess bacteria suspension, leaf discs were transferred to MS basal medium containing 100 μM acetosyringone and co-cultivated for 48 h. After co-culture explants were rinsed with sterile water, blotted dry and transferred onto hormone-free MS basal media containing 100 mg/l cefotaxime. After 4-weeks hairy roots appeared on cut ends of the explants and then they were detached and cultured onto fresh MS media. The induced roots were subcultered several times on medium containing decreasing concentrations of cefotaxime to get the bacteria free hairy root cultures lines. Hairy roots obtained from a single clone were transferred to WPM medium containing 3% sucrose and 0.3% gelrite, and incubated at 25 ± 2°C in the dark condition. The cultures were subcultured every 4-weeks and used for further analysis. Other solid media including N6 and B5 were also used and growth parameters were studied.

Apart from solid media, hairy root growth conditions were also optimized in different liquid media including MS, N6, WPM and B5. Hairy roots better line obtained from the solid WPM medium were cut (1.5 cm) and transferred to a 125 mL flask containing 20 mL of liquid B5 medium (Gamborg et al. 1968). Roots were kept in a growth chamber at 25 ± 2°C at 100 rpm rotation in the dark. The hairy roots were harvested every week for 8 weeks and their dry weight (DW) was recorded. A growth curve was plotted between time of proliferation and total mass gain by the growing hairy roots.

### Confirmations of transgenic hairy root lines

Genomic DNA was extracted from transformed hairy roots and non-transformed roots (control) of *G. scabra.* Approximately 100 mg of samples was pulverized with liquid nitrogen in a mortar pestle and then gDNA was extracted by DNeasy® Plant Mini Kit (Qiagen, Germany) and stored at 4°C. PCR mixture containing 50 ng of genomic DNA, 1 μM of oligonucleotide primers final concentration, 25 μl of 2X Taq Master Mix buffer and volume was make up to 50 μl with sterile distilled water. The PCR was performed to amplify internal *rolB* and *rolC* gene fragment (Cho et al. 1998). The first primer pair of *rolB* gene was 5′-ATG GAT CCC AAA TTG CTA TTC CCC CAC GA-3′ and 5′-TTA GGC TTC TTT CAT TCG GTT TAC TGC AGC-3′. And, the second primers for detecting the *rolC* gene was 5′-ATG GCT GAA GAC GAC CTG TGT T-3′ and 5′-TTA GCC GAT TGC AAA CTT GCA C-3′. The PCR program comprised of an initial denaturing step of 5 min at 94°C followed by 35 cycles of 45 s at 94°C, 30 s at 57°C and 45 s at 72°C and a final extension step of 10 min at 72°C. Approximately 10 μl of PCR products were electrophoresed on 1% agarose gel, stained with ethidium bromide, and visualized under UV.

### Growth regulators and secondary metabolite accumulation

Selected hairy root lines were used to study the effect of PGRs on growth and accumulation of secondary metabolites. B5 media supplemented with 1.0 mg/l concentration of NAA, thiadiazuron (TDZ), zeatin and IBA separately, were used to grow the hairy roots. The increase in total biomass and secondary metabolite content were analyzed after 4 weeks of subculture.

### High performance liquid chromatography (HPLC) analysis

The HPLC system (Hitachi) was equipped with L-2130 binary pump, an L-2200 auto-sampler and an L-2450 PDA-UV detector. The chromatographic separation of analytes was performed at ambient temperature using a Mightysil RP-18 GP column (250 × 4.6 mm, 5 μm). The auto sampler was also set at ambient temperature. Data were collected and analyzed using EZchrom Elite Version 3.13 software.

For obtaining the best separation results, the chromatographic condition of HPLC was optimized. Solvents that constituted the mobile phase consists methanol (solvent A) and 0.05% phosphoric acid in water (solvent B). The mobile phase was run with gradient elution at a flow rate of 1 ml/min. In the preliminary experiments, the elution conditions applied are as follows: 0–25 min, linear gradient 20-35% A; and, finally, reconditioning steps of the column was 20% A isocratic for 10 min.

Gentiopicroside, swertiamarin and loganic acid were purchased from National Institute for Control of Pharmaceutical and Biological Products (Beijing, PR China) for the standard. Standard solutions were prepared by dissolving 2 mg of each standard in 2 ml of methanol. Dissolved solutions were filtered through a 0.22 μm (Nalgene®, New York, USA) filter and further diluted to the concentration of 100, 50, 25, 10, 5 and 2 mg/l. Calibration curves were established based on six points covering a concentration range of 100–2 mg/l for all three standards. A 10 μl of standard solution was used for HPLC injections. Calibration graphs were plotted based on linear regression analyses of the peak areas in response to concentrations of standards injected.

The roots were harvested from the culture flask and their fresh weight was recorded. The samples were freeze-dried to determine its dry weight. The dried sample (0.1 g) was crushed into fine powder and ultra-sonicated for 10 min in 10 ml methanol: water (7:3). The supernatant was collected after centrifugation and the process repeated three times for each sample. The combined methanol/water extracts were evaporated to dryness in a rotary evaporator. The residue was dissolved in 10 ml methanol : water (1 : 1) and filtered through 0.22 μm membrane filter before analysis.

### Statistical analysis

Data were analyzed statistically by using Statistical Analysis System SAS 9.1 for ANOVA and the least significant difference (LSD) tested at 5% probability level (p ≤ 0.05). Transformation experiment was set up in a randomized design with three replicates of 15 explants each. HPLC analyses were also performed in triplicate. All data were the mean ± standard deviation (SD).

## Results and discussion

### Induction of hairy roots and molecular analysis

**Figure 1:**
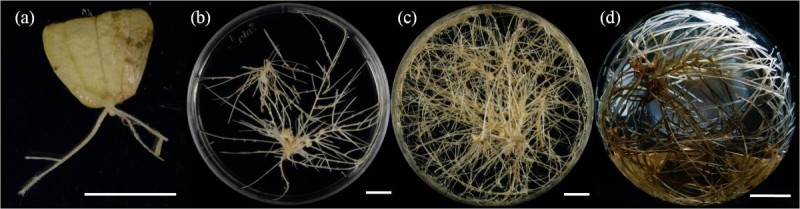
***Agrobacterium rhizogenes*** **-mediated hairy root culture in** ***Gentiana scabra*** **. (a)** Induction of hairy roots from leaves cut end **(b)** cultured on WPM solid medium 4-weeks **(c)** cultured on WPM solid medium 8-weeks and **(d)** cultured on B5 liquid medium 4-weeks. Scale bar = 1.0 cm.

**Figure 2:**
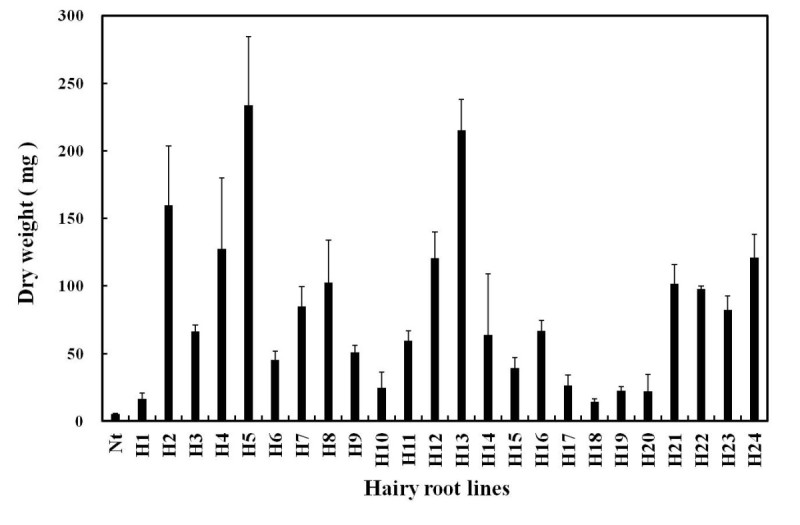
**Growth patterns of total 24 transformed hairy root clones of** ***G*** **.** ***scabra*** **on WPM solid medium compared to non-transformed (Nt) clone.** Dry weights (in mg) are of the 8 weeks old grown cultures. Values are the mean ± SD from three replicates.

Hairy root initiations were observed in 20.8% infected leaves after 4-weeks of infection with *A. rhizogenes* strains ATCC15834. First, growth of hairy roots was optimized for different solid media and it was found that WPM supported better growth. Hairy roots were isolated individually and grown on phytohormone-free WPM medium containing 100 mg/l cefotaxime to obtain bacteria free culture lines. After 3-time subculture, most hairy roots were free of bacteria and then cultured on antibiotics-free medium. All the selected lines showed proliferation in WPM culture medium (Figure 1). Considerable variations in growth capacity among individual lines were observed after the 8-weeks cultivation. Figure 2 shows dry weight gain patterns of 24 different hairy root culture lines on WPM solid medium. For example, biomass of hairy root line 5 (H5) increased from 5.3 mg to 233.6 mg (44 times), line 13 (H13) increased 40 times, line 2 (H2) increased 30 times, line 12 (H12) and line 24 (H24) increased 23 times (Figure 2). According to Cho et al. (1998) the growth variations in hairy root clones could occur because of different expression of T-DNA genes present in the transformed roots.

Several reports have documented successful induction of hairy roots in other *Gentiana* species. Momčilović et al. (1997) used *A. rhizogenes*-mediated (strains ATCC15834 or A4M70GUS) transformation of four *Gentiana* species. Hairy root lines of *G. macrophylla* were established with *A. rhizogenes* strain R1000 (Tiwari et al. 2007; Zhang et al. 2010).

Highest hairy root induction (5.6 to 33.3%) was observed in stem explants of *G. cruciata*, whereas the leaf explant provided only up to 6.7% hairy root induction (Hayta et al. 2011). Generally, leaf explants showed a very low level of transformation rates in gentian species (Mishiba et al. 2006; Tiwari et al. 2007). However, in our result we obtained 20.8% hairy root induction using leaf explants of *G. scabra* which is significantly higher from other reports on *Gentiana*. In another study, *G. macrophylla* showed 12-32% transformation rates with mature leaf as an explant source and they have also reported that bacterial strains considerably influence the transformation efficiencies (Tiwari et al. 2007).

**Figure 3:**
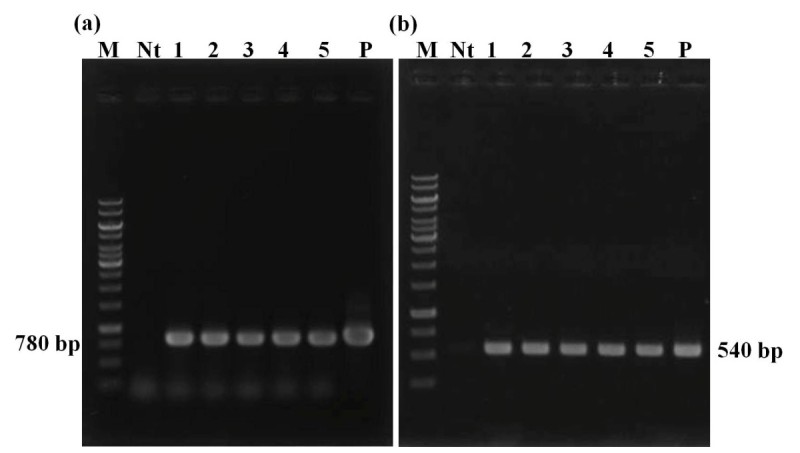
**Molecular characterization of transgenic hairy roots.** PCR confirmation of some selected transgenic hairy roots of *G. scabra* showing the amplification of **(a)** *rol B* gene amplification (780 bp) **(b)** and *rol C* gene amplification (540 bp). Lane M: Molecular weight marker; Lane Nt: non-transgenic line; Lane No. 1–5: transformed hairy root lines; P: positive control.

The hairy root lines were subjected to DNA isolation, and PCR amplified with gene specific primers to show the integration of *rolB* and *rolC* genes into the genome of *G. scabra*. The isolated DNA showed *rolB* and *rolC* gene amplification of 540 and 780 bp respectively, while no amplification was observed in non-transformed roots. Some examples are shown in Figure 3. Many studies have pointed out that hairy root metabolite production also depends on *rol* gene expression (Tiwari et al. 2007; Bulgakov 2008).

### Establishment of root liquid cultures of *G. scabra*

**Figure 4:**
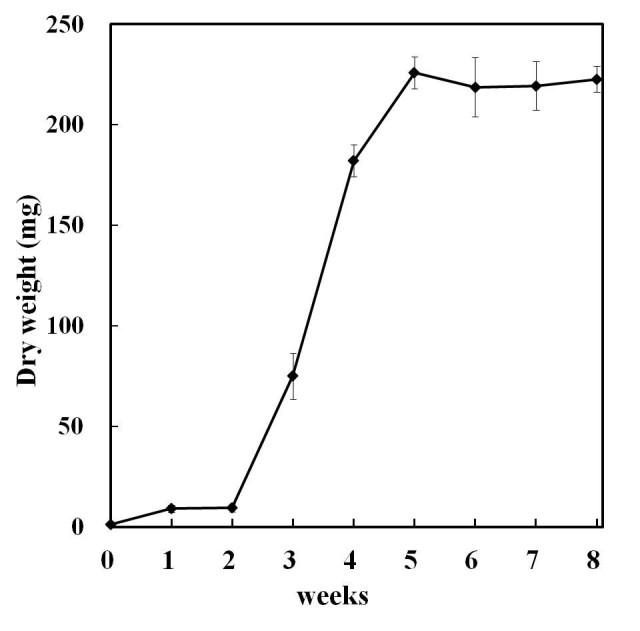
**The growth course of hairy root of** ***G*** **.** ***scabra*** **in liquid B5 medium.** Observations were recorded (dry weight in mg) after 8-weeks of culture.

To determine the best suitable media composition and culture conditions, liquid cultures of hairy root clone H5 was initiated in N6, WPM, MS and B5 media. The growth rate was calculated on (weight at the end of subculture/ inoculum weight) DW basis. We found that initial establishment of root cultures on B5 liquid media was most suitable while, other media resulted in callus formation also. The maximum DW biomass (42- fold) was achieved in B5 media composition after 8-weeks, while non-transformed root showed a slight increase in DW (Data not show). The time course for changes in dry weight of fastest growing hairy root line 5 (H5) during the 8-weeks culture period is shown in Figure 4. After 5-weeks, the dry weight increased 43 times (5.3 to 226 mg) and then stopped growing (Figure 4).

The composition and type of culture medium are known to affect the growth and proliferation of hairy roots (Giri and Narasu 2000; Murthy et al. 2008). Previous studies have shown, high organic nitrogen containing B5 medium was more suitable for hairy root growth and survival in *G. macrophyla* and *G. cruciata* (Tiwari et al. 2007; Hayta et al. 2011). Similarly we also observed a higher growth rate of hairy roots in *G. scabra* on same B5 liquid medium composition. Gamborg’s B5 vitamins have a high concentration of thiamine and it has been reported that thiamine is essential for continuous growth of *in vitro* root cultures (Jacob and Malpathak 2005).

### Metabolites accumulated during hairy root growth

**Figure 5:**
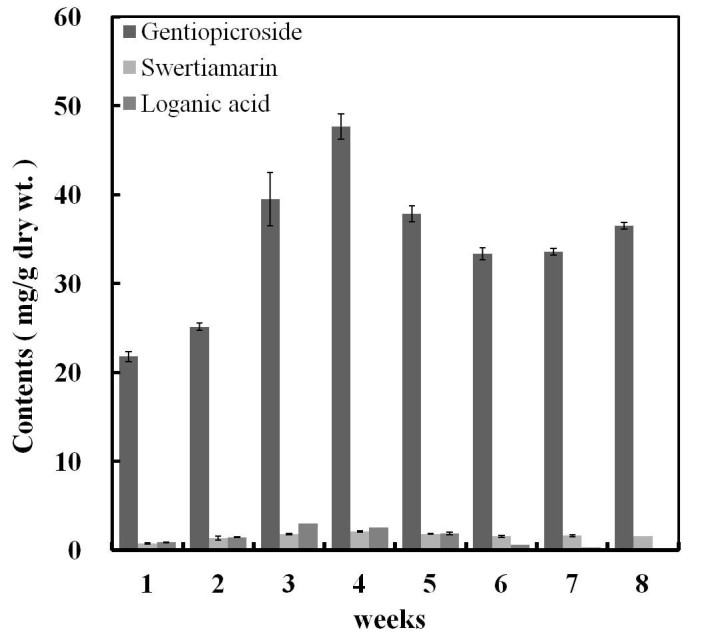
**Effect of different culture periods on the production of iridoids and secoiridoids in hairy roots of** ***G*** **.** ***scabra*** **.** Values are the mean ± SD from three replicates.

In the present study, the time course for changes we used HPLC to assess hairy root clones for metabolite products loganic acid, swertiamarin and gentiopicroside in *G. scabra* dry roots. The time course for changes in the fastest growing hairy root line 5 (H5) during the 8-weeks culture period showed the product stabilizes at the 5-weeks period. We found that loganic acid, swertiamarin and gentiopicroside contents significantly increased up to 3-weeks and reached to maximum level at 4-weeks, then the content slowly reduce. In the 4-weeks cultured roots loganic acid, swertiamarin and gentiopicroside contents were 0.78, 1.70 and 43.26 mg/g, respectively (Figure 5). The overall results showed that, the 4-weeks grown hairy root H5 cultured on B5 medium has increased (36- fold) biomass and the secondary metabolites loganic acid, swertiamarin and gentiopicroside contents. The increases in biomass were most abundant in 15–30 days in the hairy root growth of *G. macropylla*, and gentiopicroside was also maximum after 25 days of culture as compared to control plant (Tiwari et al. 2007), which are consistent with our results.

### Effects of different PGRs on biomass and metabolite accumulation

Stable transgenic lines with high multiplication rates were selected for analysis. An 36- fold (5.3 to 188 mg) increase in dry weight was observed after 4-weeks culture in PGR-free B5 medium. We investigated the effects of exogenous PGRs on the growth rate and metabolites accumulation in transformed roots of *G. scabra*. Clone H5 was cultured in medium containing various concentrations of PGRs and the results are summarized in Table 1. All the data recorded after 4-weeks of culture initiation. The results showed, there was no significant total dry weight gain (0.78-, 0.81-, 0.61- and 0.83- fold) after addition of 1.0 mg/l of IBA, NAA, TDZ or zeatin, respectively. Although PGRs have a negative influence on hairy root multiplication, the loganic acid content was 5.38-, 3.37- and 1.96- fold higher in roots grown in the presence of zeatin, IBA and NAA, respectively as compared to the control. However, loganic acid was not detected in the presence of TDZ. On the other hand, increase of 1.58- and 1. 43- fold in swertiamarin, as well as 1.28- and 1.19- fold in gentiopicroside were observed with NAA and zeatin, respectively. However, zeatin and NAA effect adversely on root dry weight biomass, but the ingredient loganic acid, sertiamarin and gentiopicroside contents were increased (Table 1). Also, as compared to roots of greenhouse grown plants loganic acid (6.6- fold) and gentiopicrosides (1.8- fold) were higher in the presence of zeatin and NAA, respectively (Tables 1 and 2).

**Table 1:** **Effect of different PGRs on hairy root production and secondary metabolite accumulation in** ***Gentiana scabra*** in liquid B5 medium*

PGR constituents (1 mg/L)	Average dry weight after 4 weeks (mg)**	Weight gain (folds)	Loganic acid (mg/g of dw)**	Sertiamarin (mg/g of dw)**	Gentiopicroside (mg/g of dw)**
0	188.67 ± 8.08 a	35.60	0.78 ± 0.11 c	1.70 ± 0.24 c	43.26 ± 2.28 b
IBA	148.00 ± 9.54 b	27.92	2.86 ± 0.09 b	1.36 ± 0.12 d	32.66 ± 1.71 c
NAA	154.00 ± 20.78 b	29.06	1.53 ± 0.17 d	2.68 ± 0.06 a	55.19 ± 0.74 a
TDZ	115.67 ± 5.86 c	21.82	ND	0.35 ± 0.01 e	10.03 ± 0.13 d
Zeatin	156.50 ± 16.26 b	29.53	4.20 ± 0.02 a	2.43 ± 0.07 b	51.88 ± 0.89 a

*B5 basal salts supplemented with 3% sucrose. Observations were recorded after 4 weeks of culture.

**Values are the means ± standard deviation (n = 3). Means within column followed by the same letter are not significantly different at the 5% probability level (p ≤ 0.05) by LSD (least significant difference) test.

dw: Dry weight.

ND: Non detectable.

**Table 2:** **Comparative analysis of secondary metabolite contents of greenhouse-grown plant and commercial herbs of** ***Gentiana scabra***

Source	Loganic acid (mg/g of dw)**	Sertiamarin (mg/g of dw)**	Gentiopicroside (mg/g of dw)**
Root from greenhouse grown plant	0.64 ± 0.06	4.42 ± 0.03	30.25 ± 0.12
*Gentiana* dried herb No. 1	4.13 ± 0.29	ND	7.90 ± 0.52
*Gentiana* dried herb No. 2	6.94 ± 0.37	1.06 ± 0.04	40.89 ± 1.78

**Values are the means ± standard deviation (n = 3). Means followed by the same letter are not significantly different at the 5% probability level (p ≤ 0.05) by LSD (least significant difference) test.

dw: Dry weight.

ND: Non detectable.

*In vitro* plant cell culture usually requires the presence of PGRs includes mainly auxins and cytokinins. The hairy roots have one characteristic that of their phenotype is rapid in PGR- free growth condition. As a result, the media used to culture hairy roots generally lacks PGRs. Even more, as demonstrated in transformed root cultures of *Datura stramonium*, cultures with NAA and kinetin caused a de-differentiation of root tissues (Ford et al. 1996). In several experiments, the de-differentiation influenced a significant decrease or even the cessation of secondary metabolite production (Fliniaux et al. 2004). In our findings, clone H5 line has a rapid accumulation of biomass and secondary metabolite; however, the PGR treatment did not enhance biomass production and tended to reduce fresh weights. Plant growth and defenses are restricted by their internal resources, and secondary metabolism often is negatively correlated with cell growth (Van Der Plas et al. 1995). In addition, hairy root cultures must strike a balance between growth processes and the production of defensive compounds.

In contrary, a more recent systematic test on the effects of different types of PGRs upon root growth and secondary metabolites showed that some of them have enhanced rapid growth or metabolite production. In the hairy roots of *Salvia miltiorrhiza* Bunge, the highest biomass was obtained with TDZ, while ABA inhibited hairy root multiplication but enhanced tanshinone accumulation (Gupta et al. 2011). In other experiments, increased ginsenosides content were observed in hybrid ginseng (*Panax ginseng × P. quinquefolium*) hairy root culture in B5 medium supplemented with individual or combined auxins (Washida et al. 2004).

### Comparative analysis of metabolites in roots from different sources

Three different active compounds (gentiopicroside, swertiamarin and loganic acid) were measured from the normal roots grown in greenhouse (2 months old) and from the two different perennial *Gentiana* dried herbs available in the market (Herb no. 1 and 2). Loganic acid was found 6.5- fold (4.13 mg/g) and 10.8- fold (6.94 mg/g) higher in the *Gentiana* dried herb no. 1 and 2, respectively. On the other hand, sertiamarin was not detected in herb no. 1, however, in herb no. 2 it was 4- fold lower than greenhouse grown plant roots. Slightly higher (1.35- fold) gentiopicroside content was recorded in *Gentiana* herb no. 2, though in the herb no. 1, 4- fold lower gentiopicroside was found as compared to green house grown plant (Table 2). In the presence of NAA, 1.4- and 2.5- fold higher gentiopicroside and swertiamarin were observed as compared to commercial *Gentiana* herb no. 2, whereas loganic acid content was lower in the presence of PGRs. Although, some metabolite contents were slightly higher in the roots of the commercial *Gentiana* herb, but development of root biomass in field growing plants under natural conditions usually takes long time. Also, the large populations of naturally or *in vitro* grown plants need to be harvested to attain the industrial requirements of roots for extraction of secondary metabolites. Thus, the use of hairy root cultures, where we can get large biomass in a short time period, could be a better choice for *in vitro* production of iridoids and secoiridoids from *G. scabra*. On the basis of above data we also concluded that the secondary metabolite contents are greatly affected by age and source of origin of the plants. So, to get stable and uniform product in less time (in our case 4-weeks), induction of hairy root cultures is a superior alternative without affecting the quality of products. Ando et al. (2007) indicated that the gentiopicroside content is strongly affected by the stage and environment of development of the plant as well as the preparation process.

## Conclusions

To the present author’s knowledge, this is the first report on the establishment of *G. scabra* hairy root cultures. The conditions for cultivation of *G. scabra* hairy roots, with regard to optimal growth and biosynthesis of secondary metabolites, were determined. Among PGRs used in the experiments, the best production of loganic acid, swertiamarin and gentiopicroside content was obtained with the use of the zeatin. On the other hand variability in iridoids and secoiridoids contents among *in vitro* grown plants and commercially available *Gentiana* herbs were also identified. Since hairy root culture is less expensive, less laborious, required less growth period and eco-friendly method, it might be a good alternative for production of important medicinal ingredients from *Gentiana* genus. The use of hairy root cultures as an alternative will not only reduce the dependence of the pharmaceutical industry on natural habitats but also ensure the quality of raw materials which are affected by various factors.

## Authors’ original submitted files for images

Below are the links to the authors’ original submitted files for images. Authors’ original file for figure 1 Authors’ original file for figure 2 Authors’ original file for figure 3 Authors’ original file for figure 4 Authors’ original file for figure 5
