# Supplementary figures and images for: Establishment of hairy root lines and analysis of iridoids and secoiridoids in the medicinal plant Gentiana scabra
Source: Bot Stud. 2014 Feb 2;55:17. doi: 10.1186/1999-3110-55-17 (PMC5430378; doi:10.1186/1999-3110-55-17)

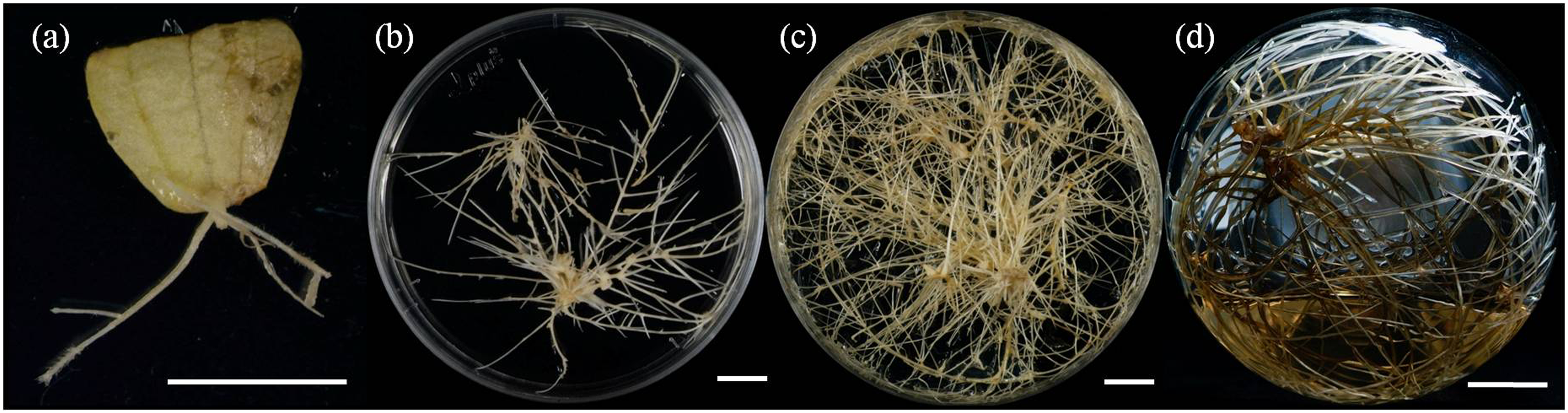

Supplement: Supplementary file 1 — Authors’ original file for figure 1 [file 40529_2013_69_MOESM1_ESM.tif]

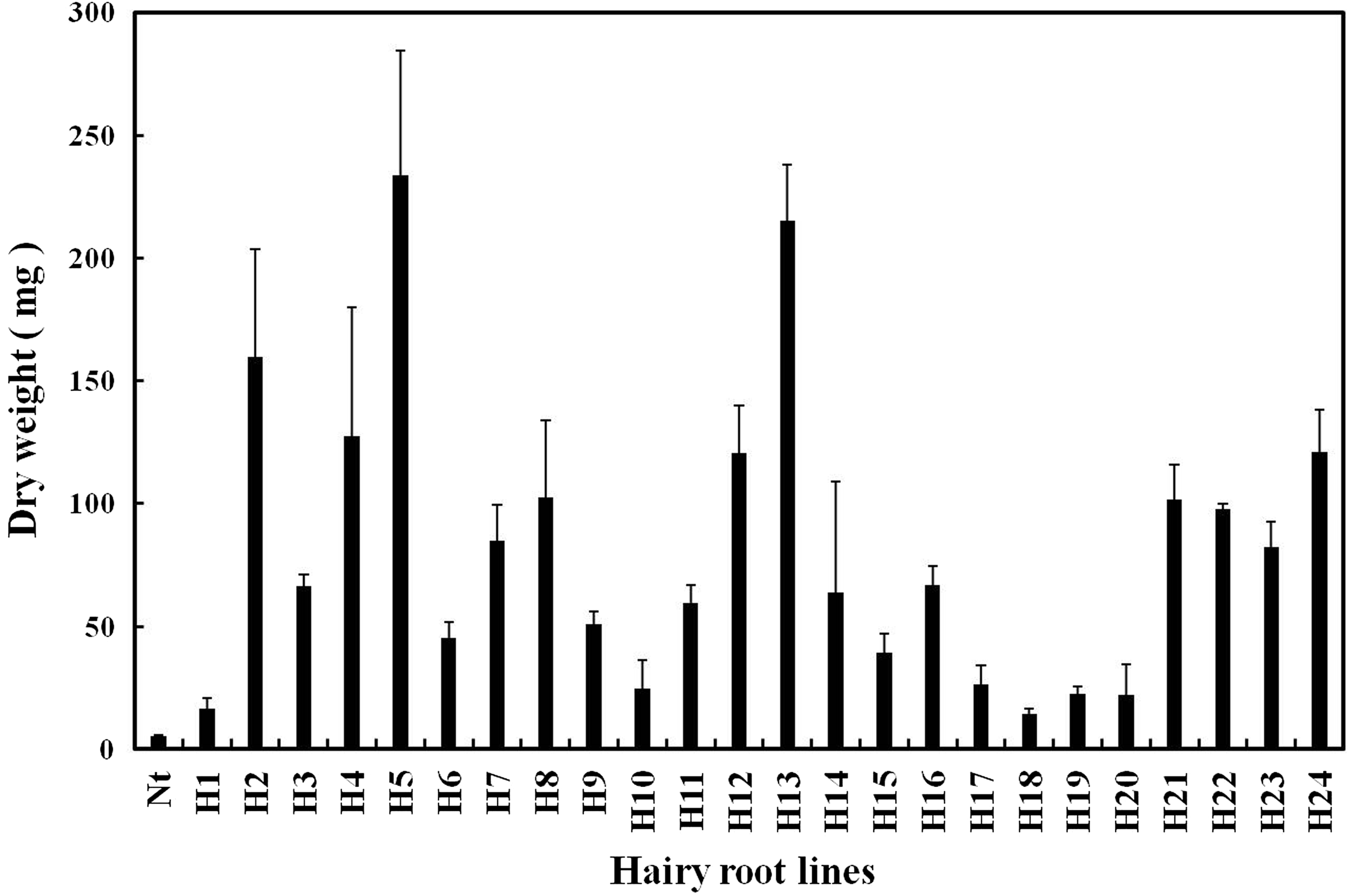

Supplement: Supplementary file 2 — Authors’ original file for figure 2 [file 40529_2013_69_MOESM2_ESM.tif]

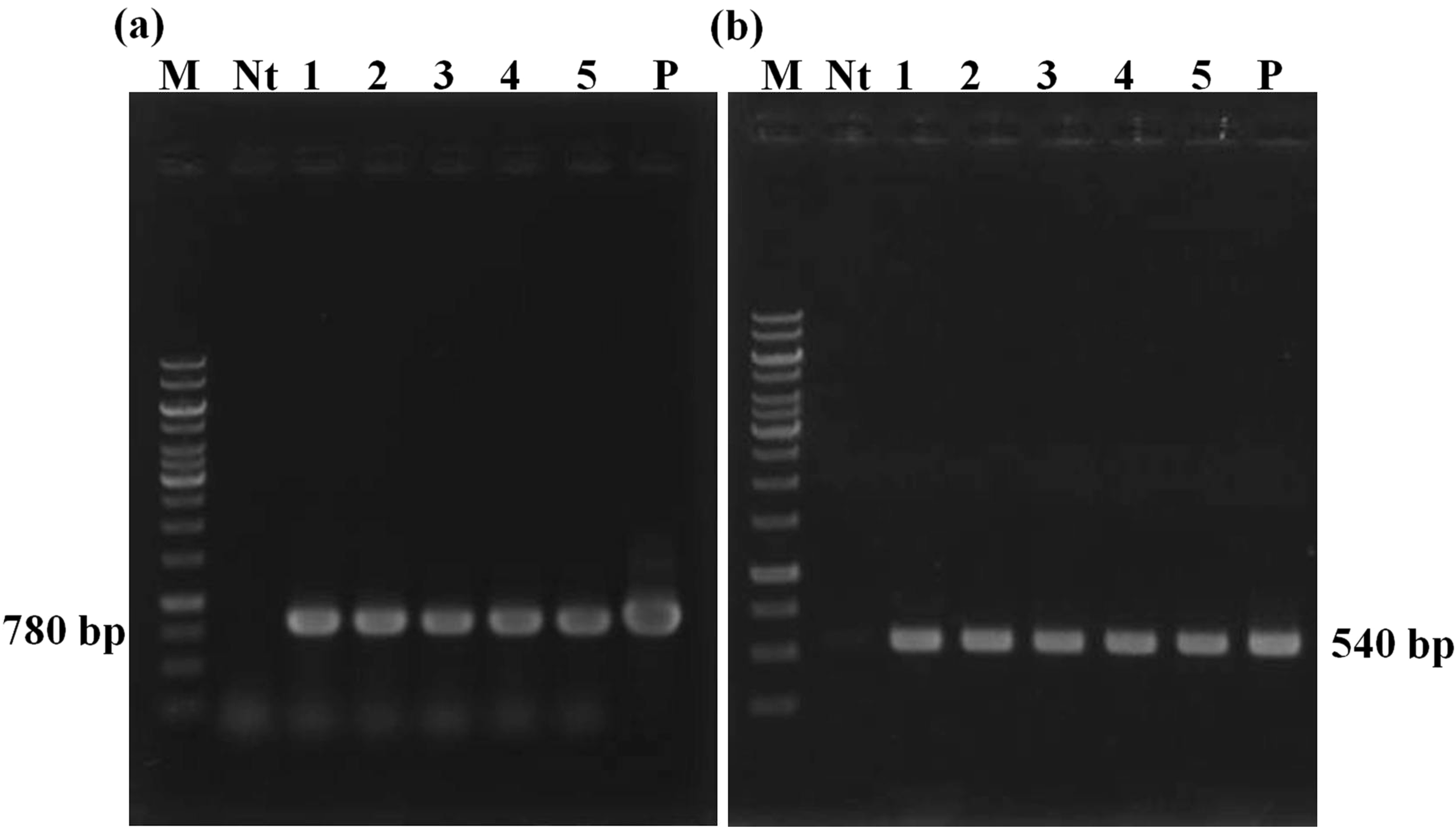

Supplement: Supplementary file 3 — Authors’ original file for figure 3 [file 40529_2013_69_MOESM3_ESM.tif]

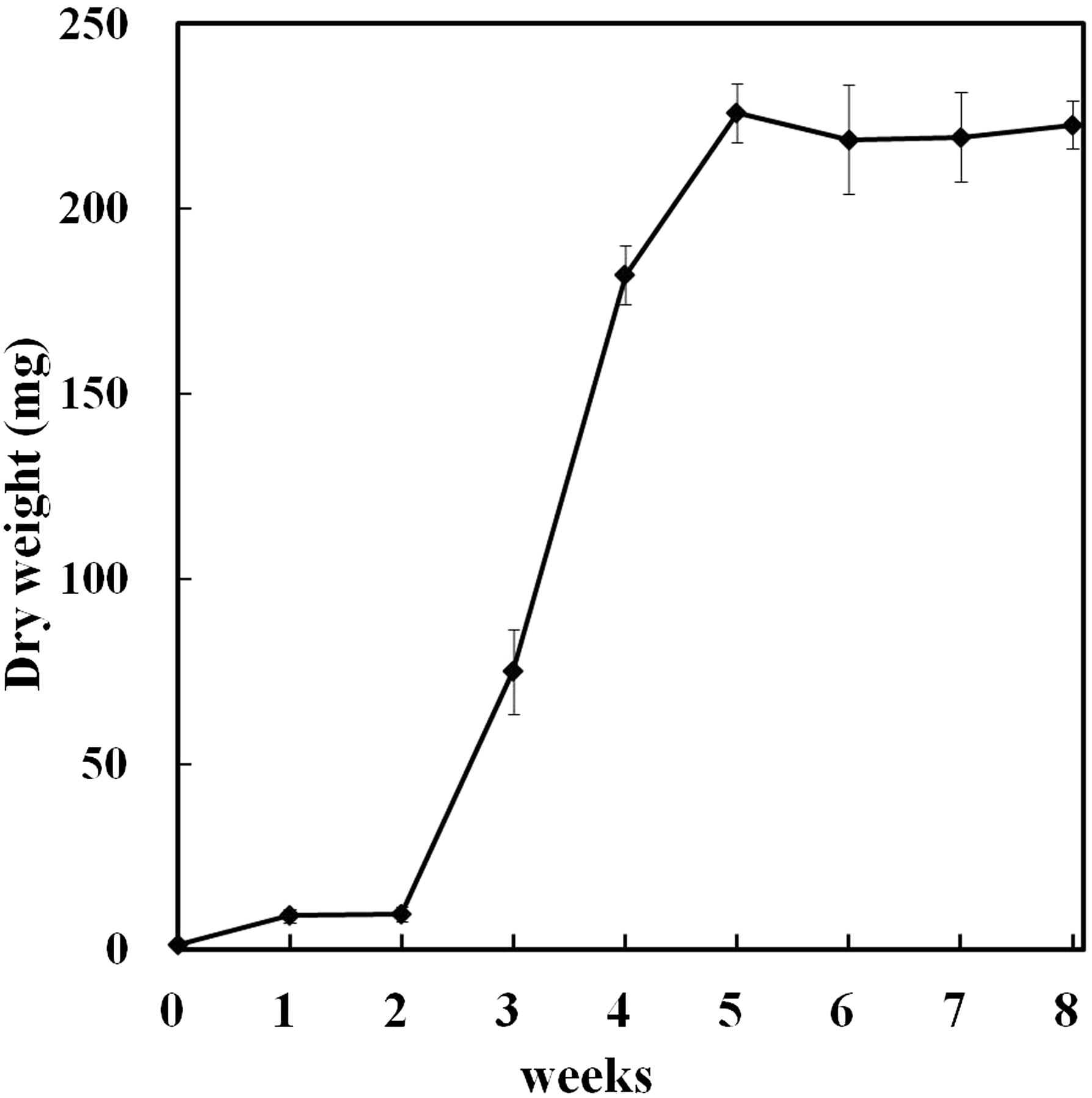

Supplement: Supplementary file 4 — Authors’ original file for figure 4 [file 40529_2013_69_MOESM4_ESM.tif]

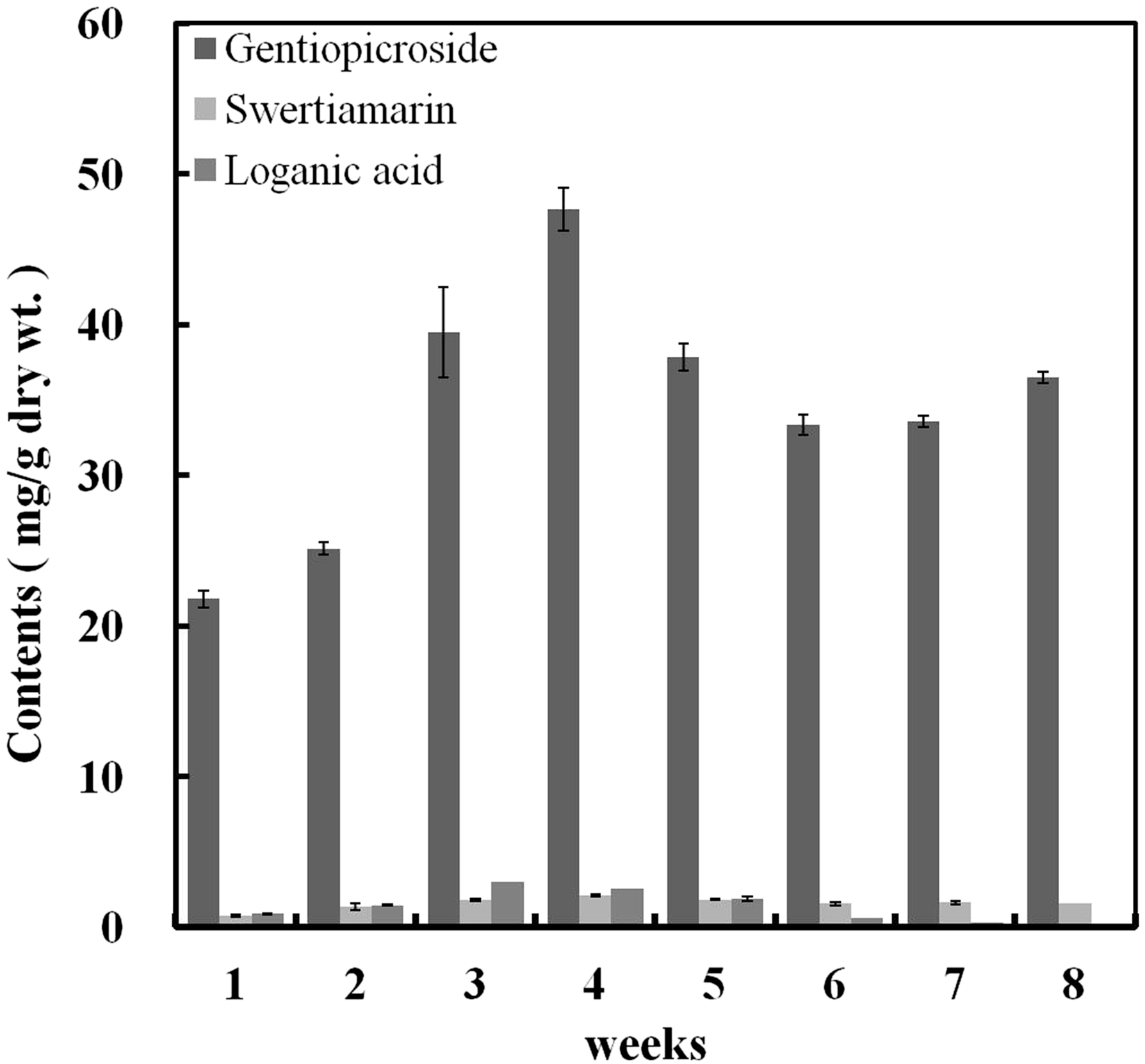

Supplement: Supplementary file 5 — Authors’ original file for figure 5 [file 40529_2013_69_MOESM5_ESM.tif]
